# Hydrogen Sulfide: A Multitarget Therapeutic for Neuroinflammation in Neurodegenerative Diseases

**DOI:** 10.34133/research.1107

**Published:** 2026-02-18

**Authors:** Yuqing Zhang, Qiong Zhang, Li Yang, Yang Fan, Peng Zheng, Tiangui Liu, Xin Gao, Zhelei Ren, Xinpeng Wang, Bowen Zhou, Wei Liu, Tao Xin, Longguang Tang, Min Han

**Affiliations:** ^1^ School of Clinical Medicine, Shandong Second Medical University, Weifang, Shandong 261053, P. R. China.; ^2^ Department of Neurosurgery, The First Affiliated Hospital of Shandong First Medical University & Shandong Provincial Qianfoshan Hospital, Jinan, Shandong 250014, P. R. China.; ^3^ Laboratory of Basic and Translational Neuromedicine, The First Affiliated Hospital of Shandong First Medical University, Jinan, Shandong 250014, P. R. China.; ^4^ The Affiliated Hospital of Shandong Second Medical University, Weifang, Shandong 261053, P. R. China.; ^5^ Department of Geriatrics, The Fifth People’s Hospital of Jinan, Jinan, Shandong 250000, P. R. China.; ^6^ College of Clinical Medicine, Jining Medical University, Jining, Shandong 272067, P. R. China.; ^7^ School of Clinical Medicine, Binzhou Medical University, Yantai, Shandong 264003, P. R. China.; ^8^ Department of Pharmacy, Center for Regenerative and Aging Medicine, The Fourth Affiliated Hospital of School of Medicine, and International School of Medicine, International Institutes of Medicine, Zhejiang University, Yiwu 322000, P. R. China.

## Abstract

Neurodegenerative diseases (NDDs) are characterized by the progressive degeneration of specific neuronal populations, remain incurable, and impose an escalating global health burden due to aging populations. While therapeutic options had expanded in recent years, their overall efficacy remained limited. Neuroinflammation emerged as a central factor in the pathogenesis of NDDs. Hydrogen sulfide (H_2_S), an endogenous gasotransmitter known for its potent anti-inflammatory properties, gained attention as a potential therapeutic agent. This review provided a comprehensive overview of the role of neuroinflammation in NDDs, elucidated the molecular mechanisms through which H_2_S exerted its anti-inflammatory effects, and discussed recent advancements and potential clinical applications. Special emphasis was placed on the modulation of glial activity, disruption of the blood–brain barrier, regulation of the gut–brain axis, and the interplay between mitochondria and inflammasomes. Furthermore, the review integrated preclinical data on dose–exposure–response relationships to define the therapeutic window of various H_2_S donors. It also explored the potential of spatial multiomics and microbiota-targeted approaches to facilitate more precise H_2_S-based interventions. These insights provide important scientific merit for elucidating the mechanisms of NDDs and hold urgent practical relevance for developing novel therapeutics to mitigate disease progression.

## Introduction

Neurodegenerative diseases (NDDs), such as Alzheimer’s disease (AD), Parkinson’s disease (PD), Huntington’s disease (HD), and amyotrophic lateral sclerosis (ALS), are chronic conditions involving progressive loss of specific neurons in the central nervous system (CNS) [1,2]. Driven by global aging, the associated disability and socioeconomic burden are rising, and NDDs are projected to become the world’s second leading cause of death by 2040 [3]. Because of poor neuronal regeneration, neurological function declines progressively. Although therapeutic modalities such as gene therapy [4] and immunotherapy [5] are advancing rapidly, their broad clinical application is currently limited by high costs, response heterogeneity, and procedural complexity [6,7]. Consequently, developing safe, accessible, and CNS-targeted therapies is urgently needed, requiring a deeper understanding of core disease mechanisms.

Chronic neuroinflammation is established as a central driver of NDD progression [8,9]. It manifests through aberrant glial activation [10,11], peripheral immune cell infiltration [12], and dysregulation of the complement system and inflammasomes [13]. Across NDDs, misfolded or aggregated proteins and synaptic dysfunction converge on innate immune sensing in microglia and astrocytes, engaging pattern recognition receptors and complement pathways that drive maladaptive synaptic pruning and chronic neuroinflammation [14]. These interconnected processes form a self-reinforcing inflammatory network. Concurrently, neurovascular unit impairment and blood–brain barrier (BBB) dysfunction facilitate peripheral immune signaling and leukocyte trafficking into the CNS. This couples systemic inflammation to mitochondrial stress and inflammasome activation, thereby sustaining a self-propagating “inflammation–mitochondria–proteostasis” vicious cycle that leads to irreversible neurodegeneration [1,13]. Consequently, targeting molecules capable of modulating both neuroimmune responses and mitochondrial homeostasis is considered a promising therapeutic strategy.

Gasotransmitters, including nitric oxide (NO), carbon monoxide (CO), and hydrogen sulfide (H_2_S), are endogenous mediators that freely diffuse across membranes to regulate diverse physiological processes [15]. Among them, H_2_S has garnered considerable attention for its potent antioxidant and anti-inflammatory properties, particularly in neuroprotection [16]. Experimental studies have shown that H_2_S alleviates pathological features of AD, PD, and HD [8,17–19]. Unlike many therapeutics, H_2_S possessed high lipid solubility, facilitating its penetration of the BBB [20]. By modulating endogenous protective pathways, H_2_S represents a promising target for treating NDDs with high translational potential [21,22].

While previous reviews have summarized H_2_S signaling in NDDs [23–26], they primarily focused on neuronal cascades or global redox regulation, often overlooking glial biology, BBB dysfunction, and advanced drug-delivery strategies. Furthermore, few studies have synthesized dose–effect relationships, safety profiles, or gut–brain axis interactions [27–29]. Consequently, the literature offers limited guidance for the rational design of translatable H_2_S interventions.

**Fig. 1.**
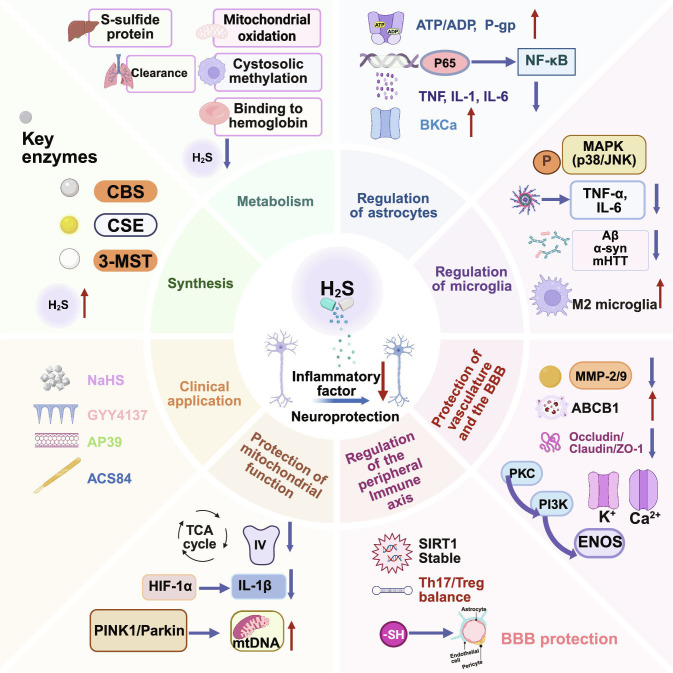
Therapeutic potential of H_2_S as a multitarget agent in neuroinflammation. H_2_S acts as a versatile gasotransmitter that modulates glial activation, vascular integrity, mitochondrial function, and the gut–brain axis. By suppressing maladaptive neuroinflammatory responses, H_2_S provides a mechanistic framework for the rational design of targeted interventions in neurodegenerative diseases (created with BioRender).

This work systematically reviewed the molecular mechanisms of H_2_S-mediated neuroprotection, positioning H_2_S as a multitarget modulator of the neuroinflammatory network-encompassing glial phenotypes, BBB integrity, and mitochondria–inflammasome crosstalk. We analyzed how H_2_S influenced neurovascular stability to impact NDD progression Fig. 1. Additionally, we synthesized preclinical data to delineate therapeutic windows for various H_2_S donors and examined emerging strategies, such as nano-delivery systems and gut–brain axis modulation, to enhance brain targeting [30–32]. This review bridges the translational gap by elucidating the pleiotropic mechanisms of H₂S, providing a robust theoretical framework for developing next-generation disease-modifying therapies targeting neuroinflammation.

## Methods

### Literature search and evidence synthesis

We conducted a comprehensive literature searched in PubMed, Web of Science, and Scopus (up to November 2025) to identify studies exploring H₂S modulation in NDDs. Search queries combined terms for H₂S donors/biology (“hydrogen sulfide” and “persulfidation”), neuroinflammation targets (“microglia”, “NOD-, LRR- and pyrin domain-containing protein 3” [NLRP3], and “nuclear factor kappa-light-chain-enhancer of activated B cells” [NF-κB]), and major NDDs (“Alzheimer’s”, “Parkinson’s”, “amyloid”, and “α-synuclein”) that were constructed based on predefined criteria. To ensure methodological rigor and reproducibility, the screening process was conducted based on predefined criteria. We prioritized primary experimental and translational studies that (a) utilized established in vitro or in vivo NDD models; (b) applied defined H₂S modulators (including donors, prodrugs, or delivery systems) with reported dosage regimens; and (c) assessed specific neuroinflammatory or neurodegenerative endpoints. Exclusion criteria included non-English publications, conference abstracts without full data, and studies using nonspecific sulfur compounds.

Following an initial screening of titles and abstracts, full-text assessments were conducted, yielding 163 studies for qualitative synthesis. Data were systematically extracted using structured tables to catalog donor chemistry, dosing regimens, model types, and outcomes. Given the substantial heterogeneity in experimental designs (e.g., diverse H₂S donors and administration routes) and the paucity of standardized quantitative brain exposure data, formal meta-analysis was precluded. Consequently, a semiquantitative synthesis was employed to evaluate cross-study consistency and dose–effect relationships.

Limitations of this methodology include the restriction to English-language databases, which may exclude relevant regional studies, and the inherent difficulty in directly comparing effective doses across different H₂S release mechanisms without unified pharmacokinetic standardization.

## Hydrogen Sulfide Synthesis, Metabolism, and Homeostasis

### Biosynthetic pathways of hydrogen sulfide

Endogenous generation of H_2_S occurred through both enzymatic and nonenzymatic pathways [33,34]. The enzymatic routes relied on 3 key enzymes: cystathionine β-synthase (CBS), cystathionine γ-lyase (CSE), and 3-mercaptopyruvate sulfur transferase (3-MST) [35]. CBS and CSE utilized L-cysteine or homocysteine as substrates to produce H_2_S in a pyridoxal-5′-phosphate (PLP)-dependent manner [36,37], whereas 3-MST catalyzes H_2_S generation from 3-mercaptopyruvate (3-MP) via a PLP-independent mechanism [38,39]. In the CNS, CBS and 3-MST were co-expressed and acted synergistically to regulate the dynamic balance of H_2_S, thereby maintaining its physiological functions [40]. Thus, modulation of the expression and posttranslational modifications of H_2_S-synthesizing enzymes represented a critical approach to restoring H_2_S levels under pathological conditions [41–43].

In addition, H_2_S could also be generated through nonenzymatic reactions. In erythrocytes or microenvironments enriched in transition metals, reduced sulfur species could slowly release H_2_S under catalytic or pH-favorable conditions [44]. The gut microbiota also played a major role in H_2_S production by fermenting sulfur-containing amino acids; in particular, sulfate-reducing bacteria generated H_2_S, which could be absorbed through the intestinal mucosa into systemic circulation [45]. Newly synthesized H_2_S might have acted directly as a gaseous signaling molecule or been converted into bound sulfur, which could be mobilized to release H_2_S in response to oxidation–reduction stimuli [46]. This dynamic “synthesis–storage–release” system underpinned the spatiotemporal specificity of H_2_S action in immune regulation, mitochondrial function, and inflammasome activation.

### Metabolism and homeostatic maintenance of hydrogen sulfide

**Fig. 2.**
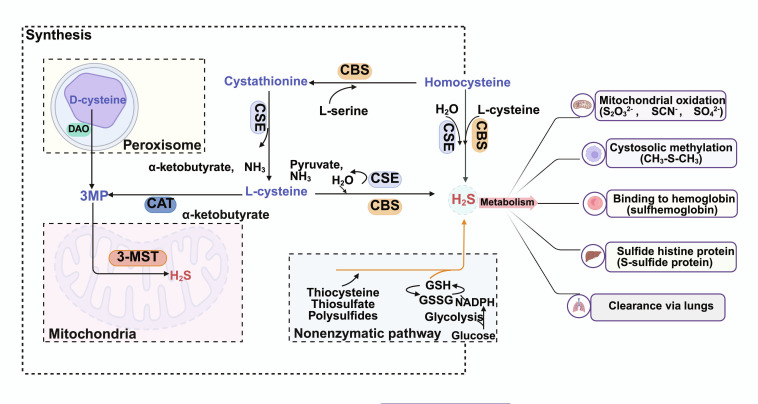
Biosynthesis, buffering, and clearance pathways that maintain H_2_S homeostasis. Endogenous H_2_S is generated primarily via enzymatic transsulfuration, in which CBS and CSE use L-cysteine and/or homocysteine as substrates. A complementary route involves 3-MP sulfurtransferase (3-MST), which utilizes 3-MP produced by cysteine aminotransferase (CAT) or D-amino acid oxidase (DAO) (including peroxisomal D-cysteine metabolism). In addition to enzymatic production, H_2_S can be slowly released from nonenzymatic reactions and from sulfane–sulfur reservoirs (e.g., persulfides, polysulfides, and thiosulfate), which buffer local availability. Clearance occurs predominantly through mitochondrial oxidation, initiated by sulfide: quinone oxidoreductase (SQOR) and followed by sequential metabolism involving ethylmalonic encephalopathy protein 1 (ETHE1; persulfide dioxygenase), thiosulfate sulfurtransferase (TST), and sulfite oxidase (SUOX), yielding products such as thiosulfate and sulfate. Additional fates include cytosolic methylation and reversible binding/persulfidation of hemoglobin and protein thiols; a fraction of volatile sulfur metabolites is eliminated via the lungs (created with BioRender).

Precise control of H_2_S bioavailability is essential to preserve signaling while avoiding toxicity [47]. In mammalian cells, mitochondrial oxidation is the dominant clearance route, complemented by cytosolic methylation and reversible sequestration within sulfane–sulfur pools and protein thiols (Fig. 2) [48–50]. H_2_S homeostasis therefore reflects a dynamic balance between production, buffered storage, and clearance: when H_2_S rises, catabolic capacity and buffering increase to prevent accumulation, whereas reduced availability can be compensated by mobilization of bound-sulfur reservoirs and up-regulation of biosynthetic pathways [51–54]. Together, these mechanisms maintain local, context-dependent H_2_S/polysulfide levels that shape neuroinflammatory signaling and cellular stress responses.

## Neuroinflammation in NDDs

**Fig. 3.**
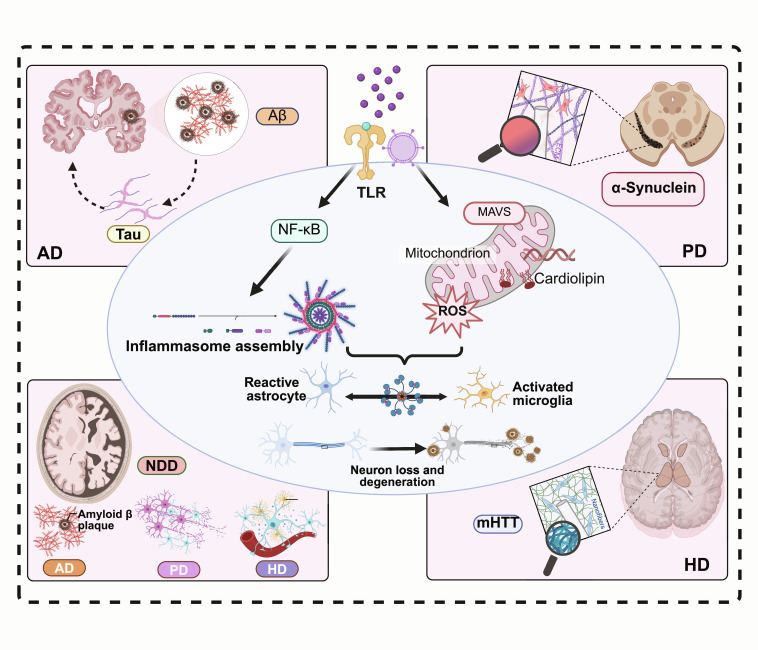
Convergent initiation and feed-forward amplification of neuroinflammation in NDDs. Disease-defining proteinopathies (e.g., Aβ/tau in AD, α-syn in PD, and mutant huntingtin in HD) and associated cellular stress act as damage-associated molecular patterns that engage innate immune sensing (e.g., TLRs and mitochondria-linked signaling such as mitochondrial antiviral signaling protein [MAVS]), leading to NF-κB activation. In parallel, mitochondrial perturbation generates mtROS and related danger signals that promote inflammasome assembly (exemplified by NLRP3), thereby amplifying cytokine-driven inflammatory outputs. These pathways reinforce microglial activation and reactive astrocyte programs, forming a self-propagating inflammatory loop that contributes to neuronal dysfunction and degeneration (created with BioRender).

NDDs were characterized by a chronic, low-grade, and persistent activation state, involving multiple pathological events such as abnormal protein aggregation, glial cell interactions, BBB disruption, and cellular homeostatic imbalance, all of which were closely associated with disease-specific molecular alterations (Fig. 3).

### Triggers of neuroinflammation in NDDs

Neuroinflammation was a key driver of the onset and progression of NDDs, and its chronic, low-level activation was closely linked to neuronal injury and disease exacerbation [55]. Abnormal protein aggregates in NDDs, including amyloid-β (Aβ), tau protein, α-syn, and mutant huntingtin protein (mHTT) [56,57], acted as damage-associated molecular patterns (DAMPs). These DAMPs were recognized by pattern recognition receptors on microglia and astrocytes, thereby activating the downstream NF-κB signaling pathway [58,59]. Under the influence of secondary activating signals, such as mitochondrial ROS (mtROS), ATP-P2X7-mediated K^+^ efflux, and cathepsin B release, the NLRP3 inflammasome was assembled and activated. This process promoted caspase-1-dependent maturation and release of interleukin(IL)-1β and IL-18, ultimately amplifying the inflammatory response and neurotoxicity [60,61]. This DAMP–NF-κB priming and secondary signaling–NLRP3 axis constituted a central upstream hub in the neuroinflammatory signaling network.

At the receptor and signaling level, multiple pattern recognition receptor families contribute to this priming/activation cascade, including Toll-like receptors (e.g., TLR2/4), receptor for advanced glycation end products, scavenger receptors, and cytosolic sensors that couple to adaptor pathways such as MyD88/TRIF and MAVS. These pathways converge on NF-κB and interferon-regulatory programs, shaping cytokine/chemokine outputs and microglial recruitment dynamics. Importantly, inflammasome activation is not an isolated module: it is tightly interwoven with mitochondrial stress signaling (mtROS and mitochondrial DNA release), autophagy/mitophagy capacity, and metabolic rewiring, collectively determining whether inflammatory responses resolve or become chronically self-sustaining [58,62].

### Roles of glial cells in inflammatory regulation in NDDs

Microglia and astrocytes were the principal effector cells of central neuroinflammation. Activated microglia exhibited functional polarization, which was commonly categorized into the proinflammatory M1 phenotype and the anti-inflammatory and reparative M2 phenotype [62]. M1 microglia up-regulated proinflammatory mediators such as tumor necrosis factor-α (TNF-α), IL-1β, IL-6, inducible nitric oxide synthase (iNOS), and cyclooxygenase-2 (COX-2) thereby exacerbating neuronal injury [60,61]. In contrast, M2 microglia promoted inflammation resolution and tissue repair by producing IL-10, tumor growth factor-β (TGF-β), and arginase-1 (Arg1) [63].

While the M1/M2 framework is useful for conceptualization, transcriptomic studies indicate a spectrum of microglial activation states in NDDs, including disease-associated microglia that coordinate lipid handling, phagocytosis, and inflammatory signaling around pathological lesions. Similarly, reactive astrocytes exist as heterogeneous states that can either support neuronal survival or amplify complement-mediated synapse loss and excitotoxicity. These state transitions are governed by local cytokine milieus (e.g., TNF, IL-1 family, and type I interferons), neuron–glia signaling, and neurovascular cues, highlighting the need to consider cell-state specificity when targeting neuroinflammation [58,59,62].

Astrocytes also displayed phenotypic plasticity. A1 astrocytes (proinflammatory phenotype) secreted complement proteins (e.g., C3), proinflammatory mediators (TNF-α and IL-1α), and chemokines (CXCL10 and CCL2), thereby contributing to synaptic phagocytosis and neuronal injury, and amplifying neuroinflammation [64]. A2 astrocytes (neuroprotective phenotype), in contrast, expressed high levels of brain-derived neurotrophic factor (BDNF), glial cell line-derived neurotrophic factor (GDNF), and S100 Calcium-Binding Protein Beta (S100β), exerting protective effects on neurons [65]. Microglia regulated astrocytic phenotype conversion by reshaping the immune microenvironment, where M1 microglial cytokines activated proinflammatory A1 astrocytes, exacerbating synaptic elimination and neuroinflammation, while M2 cytokines promoted protective A2 astrocytes, reducing inflammation and supporting neuronal survival [66]. Such glial cell interactions expanded local stimuli into network-level inflammatory responses and formed a positive feedback loop with the NLRP3-IL-1β/IL-18 axis, rendering neuroinflammation persistent in NDDs.

### BBB disruption and peripheral immune cell infiltration

During neuroinflammation, proinflammatory cytokines and matrix metalloproteinases (MMP-2/9) compromised BBB integrity, leading to down-regulation of tight junction proteins (claudin-5, occludin, and ZO-1), enhanced caveolin-1-dependent endothelial transcytosis, and basement membrane degradation [67]. These changes increased BBB permeability, allowing peripheral immune cells such as C-C chemokine receptor type 2 (CCR^2+^) monocytes and neutrophils to infiltrate the brain parenchyma. Once recruited, these infiltrating cells interacted with resident glia, activating complement and chemokine networks that propagated local inflammation into a diffuse, systemic inflammatory state, thereby accelerating neurodegeneration [68]. The BBB–peripheral immune coupling mechanism thus provided potential targets for both central and peripheral interventions.

### Neuroinflammation and cellular homeostatic imbalance

Chronic neuroinflammation induced mitochondrial dysfunction, inhibition of the autophagy–lysosomal pathway, and impaired clearance of abnormal proteins, thereby creating a vicious cycle among “inflammation–metabolism–proteostasis” that further accelerated disease progression [69,70]. Emerging evidence also highlighted the involvement of novel inflammatory cell death mechanisms, including activation of the cyclic GMP-AMP synthase-STimulator of INterferon genes (cGAS-STING) pathway and pyroptosis mediated by gasdermin D/ gasdermin E (GSDMD/GSDME) in NDDs.

At the subcellular level, inflammatory signaling and homeostatic collapse are linked through immunometabolic remodeling. Activated glia often shift toward glycolysis and altered tricarboxylic acid cycle flux, which can promote succinate/ROS accumulation and reinforce proinflammatory gene expression. In parallel, impaired mitophagy and lysosomal dysfunction reduce the clearance of damaged mitochondria and aggregated proteins, further feeding inflammasome activation and programmed cell death pathways. Thus, mitochondrial quality control and inflammatory execution programs (including inflammasome-driven pyroptosis and PANoptosis) form a mechanistic bridge between chronic neuroinflammation and irreversible neurodegeneration [71,72].

### Microbiota-derived H_2_S and the gut–brain axis in neuroinflammation

Recent studies showed that systemic inflammation and the gut–brain axis critically modulated neuroinflammation in NDDs. Neuroinflammatory changes influenced gut physiology and microbial metabolism, thereby altering the production of key microbial metabolites, including short-chain fatty acids, tryptophan-derived indoles, and sulfur-containing compounds. These metabolites shaped microglial maturation, astrocytic reactivity, and BBB integrity, establishing a bidirectional communication loop between the brain and the gut. Microbiota-derived H_2_S, mainly produced by sulfate-reducing bacteria such as *Desulfovibrio* species, exerted context-dependent effects: at physiological levels, it contributed to the maintenance of the mucosal barrier and redox homeostasis, whereas excessive H_2_S had been implicated in promoting α-syn aggregation, TLR4-dependent inflammation, and neurodegeneration in PD models [73,74]. Recent reviews suggested that rebalancing microbiota-derived H_2_S signaling through dietary interventions, probiotics, or targeted modulation of sulfur-metabolizing bacteria might be a complementary strategy to tune systemic and CNS inflammation in NDDs [75]. These observations suggested that microbiota-targeted approaches could be leveraged as a relatively noninvasive means to indirectly modulate H_2_S signaling and neuroinflammatory tone in susceptible individuals.

Collectively, neuroinflammation in NDDs represented a multistep, network-like pathological process that encompassed DAMP-triggered innate immune responses, glial phenotype switching and crosstalk, BBB disruption with peripheral immune infiltration, and mitochondrial/autophagic dysfunction. Elucidating these pathways and their interactions not only advanced our understanding of NDD pathogenesis but also identified theoretical bases and potential targets for therapeutic intervention. Recent advances in spatial transcriptomics and spatially resolved multiomics had begun to map glial subpopulations and immune niches at cellular resolution in NDD brains, revealing disease-specific microglia–astrocyte crosstalk around amyloid plaques and neurodegenerative lesions [76]. These technologies provided powerful tools to dissect how H_2_S-modulated pathways reshape local neuroinflammatory microenvironments.

## Anti-Inflammatory Effects of Hydrogen Sulfide in NDDs

**Fig. 4.**
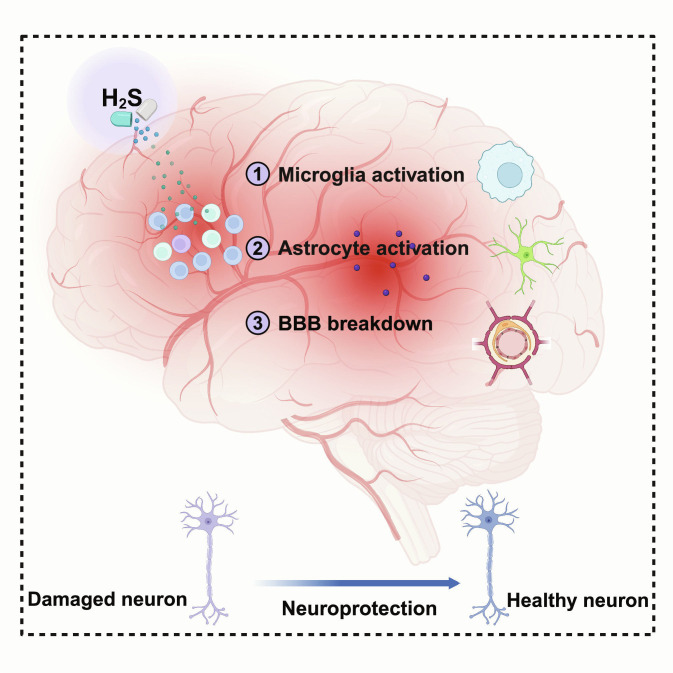
Multimodal anti-neuroinflammatory and neuroprotective actions of H_2_S in NDDs. H_2_S exerts coordinated anti-inflammatory effects by acting on 3 coupled control nodes: microglia (e.g., limiting TLR4-NF-κB signaling [60,84]), astrocytes (e.g., restraining C3-high neurotoxic programs [65,82,83]), and BBB integrity (e.g., preserving tight-junction function [67,128,129]). Collectively, these actions dampen feed-forward inflammatory toxicity, including inflammasome-linked cytokine amplification, and thereby support neuronal survival [61,147] (created with BioRender).

H_2_S exerts multilayer anti-neuroinflammatory actions by coordinately reprogramming glial states and preserving neurovascular integrity, thereby reshaping the inflammatory milieu that drives neuronal vulnerability in NDDs. As summarized in Fig. 4, these effects converge on 3 coupled control nodes—microglial activation, astrocyte reactivity, and BBB stability—providing an integrated framework for the mechanistic sections that follow [77].

### Immunoregulatory effects of hydrogen sulfide

During the neuroinflammatory process, H_2_S suppressed proinflammatory programs and enhanced the resolution of inflammation, inducing glial and peripheral immune cells to shift from a proinflammatory phenotype toward a repair/anti-inflammatory phenotype, thereby markedly reducing the intensity and duration of inflammation. Mechanistically, this involves S-sulfhydration of proteins, which regulated the activity and interactions of target proteins [78], or modulation of ion channels (K-ATP and B-KCa) that influence Ca^2+^ signaling and membrane potential [79], thereby promoting phenotype switching. In addition, H_2_S participated in electron transfer via the mitochondrial SQOR→coenzyme Q (CoQ) pathway and activated the nuclear factor erythroid 2-related factor 2 (Nrf2) pathway, leading to the up-regulation of antioxidant gene expression and protection against immune cell death [80]. Recent studies also suggested that H_2_S may have enhanced anti-inflammatory capacity through the regulation of immunometabolic reprogramming [81].

#### Modulation of astrocytic phenotypic transition

**Fig. 5.**
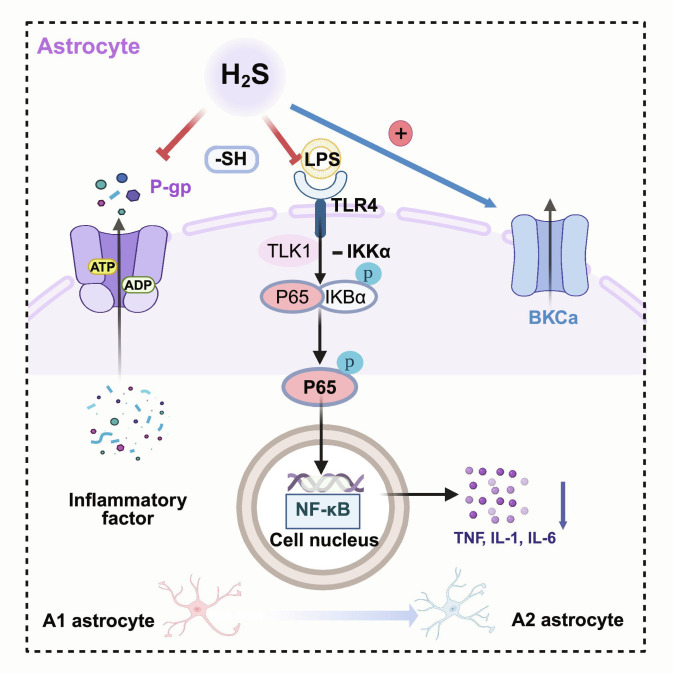
H_2_S promotes astrocytic switching from A1 to A2 programs by coupling anti-inflammatory and trophic signaling. In reactive astrocytes, H_2_S can modulate sulfhydration-sensitive targets (e.g., P-glycoprotein [P-gp]) and attenuate TLR4-NF-κB signaling (e.g., limiting p65/RelA nuclear activity), thereby reducing proinflammatory outputs that reinforce A1-like states. In parallel, H_2_S can activate BKCa channels to stabilize Ca^2+^-dependent signaling and engage trophic transcriptional programs (e.g., STAT3/CREB), facilitating a shift toward neuroprotective A2-like profiles (created with BioRender).

As summarized in Fig. 5, H_2_S promotes A1-to-A2 astrocytic switching by concurrently suppressing proinflammatory NF-κB signaling and enhancing Ca^2+^-dependent trophic transcriptional programs. H_2_S promoted the transition of astrocytes from the proinflammatory A1 phenotype to the neuroprotective A2 phenotype through 2 mechanisms. First, H_2_S suppressed the NF-κB signaling pathway by inhibiting IKKβ activity, stabilizing IκBα, and potentially S-sulfhydrating key cysteine residues of the p65 (RelA) subunit, thereby blocking its nuclear translocation and DNA binding capacity. This resulted in the down-regulation of proinflammatory cytokines such as TNF-α, IL-1β, and IL-6, effectively repressing A1-associated transcriptional programs [82]. Second, H_2_S activated large-conductance Ca^2+^-activated K^+^ channel (BKCa) channels (encoded by KCNMA1), improved intracellular Ca^2+^ homeostasis, and subsequently activated trophic transcriptional pathways such as signal transducer and activator of transcription 3/cAMP response element-binding protein (STAT3/CREB). This led to the up-regulation of BDNF, GDNF, and S100β, thereby promoting A1-to-A2 switching, enhancing synaptic support, and maintaining metabolic homeostasis [83].

#### Control of microglial phenotypic polarization

**Fig. 6.**
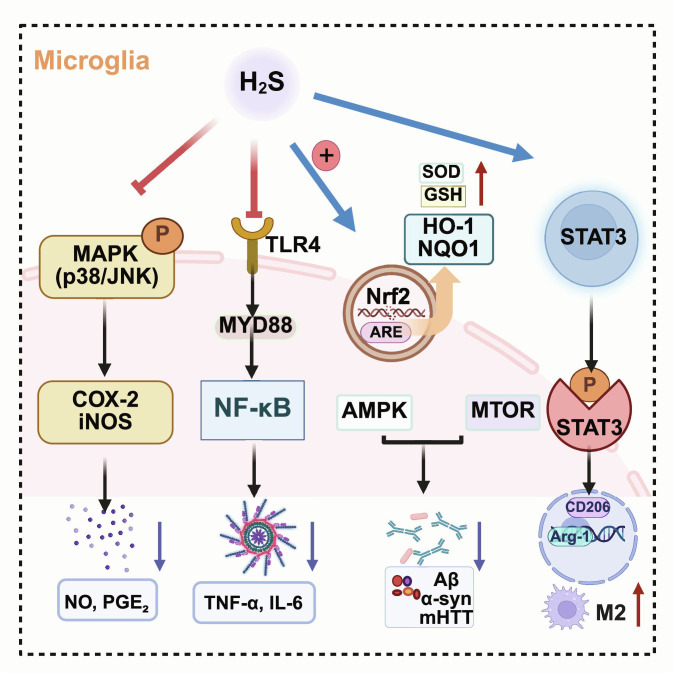
H_2_S promotes microglial phenotypic reprogramming through convergent inflammatory and stress-response pathways. H_2_S can limit proinflammatory microglial activation (e.g., restraining TLR4-NF-κB and p38/JNK MAPK signaling), engage metabolic/stress-adaptation programs that favor reparative polarization (e.g., AMP-activated protein kinase [AMPK]–mechanistic target of rapamycin [mTOR]–STAT3 axis), and enhance antioxidant defenses (e.g., Nrf2-antioxidant response element [ARE] signaling). These coordinated actions support a shift toward M2-like profiles and improved clearance capacity for neurotoxic protein aggregates (Aβ, α-syn, and mHTT) (created with BioRender).

H_2_S has been reported to reprogram microglial inflammatory states by concurrently dampening proinflammatory transcriptional programs and enhancing cellular clearance functions (e.g., autophagy/phagocytosis), thereby facilitating a shift from M1-like to M2-like profiles in NDD-relevant models [84]. As shown in Fig. 6, H_2_S decreases representative proinflammatory outputs while increasing M2-associated markers such as Arg1 and CD206 [85]. In addition, several studies suggest that H_2_S can attenuate inflammasome-linked amplification (e.g., reduced NLRP3/IL-1β signaling) in a context-dependent manner, including via upstream checkpoint modulation [86,87]. Together, these findings support H_2_S as a multitarget immunomodulator that may limit feed-forward neuroinflammation while improving microglial proteostasis control.

#### Regulation of the peripheral immune axis

H_2_S via S-sulfhydration suppressed p65-driven proinflammatory transcriptional activity and thereby reduced macrophage M1 polarization and TNF-α/IL-1β release [88]. Moreover, by regulating the glucose transporter 1 (GLUT1)–glycolysis pathway and pyruvate kinase M2 (PKM2) conformational state, H_2_S promoted immunometabolic reprogramming and alleviated secondary BBB damage [89,90]. In adaptive immunity, H_2_S up-regulated TGF-β expression in dendritic cells, directly suppressed T helper 17 cell differentiation, and promoted Treg expansion, thereby shifting the T helper 17 cell/Treg balance toward immunosuppression and mitigating the pathogenic IL-17 axis in amplifying neuroinflammation [91].

### Hydrogen sulfide protection of vasculature and the BBB

**Fig. 7.**
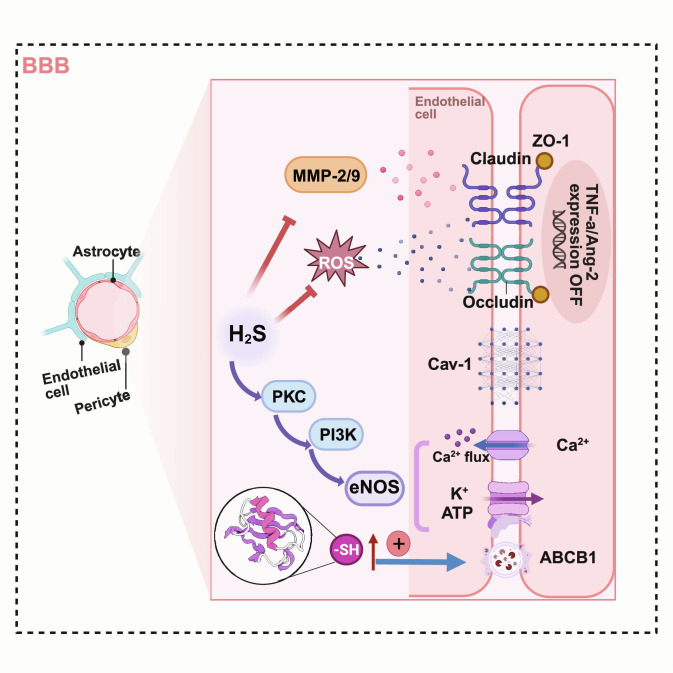
Multitarget BBB-protective actions of H_2_S in neuroinflammation. H_2_S supports BBB integrity by converging on endothelial barrier structure and function: it limits oxidative/protease-driven junctional injury (e.g., reduced ROS and MMP-2/9 activity, preserving tight-junction function) [67,128,129], promotes endothelial signaling that maintains microvascular homeostasis (e.g., protein kinase C [PKC]/phosphoinositide 3-kinase [PI3K]–Akt-eNOS axis and NO-H_2_S crosstalk) [93,94], and modulates transport and permeability pathways (e.g., S-sulfhydration-sensitive regulation of ABCB1/P-gp and suppression of caveolin-1-dependent transcytosis) [94,95]. Together, these actions are consistent with reduced BBB leakiness and dampened amplification of neuroinflammatory signaling (created with BioRender).

H_2_S preserves neurovascular integrity and mitigates BBB leakiness, thereby weakening the feed-forward loop linking barrier failure, peripheral inflammatory signaling, and sustained neuroinflammation [92]. As summarized in Fig. 7, BBB protection by H_2_S can be conceptualized as a coordinated program that (a) stabilizes endothelial junctional and transport functions while restraining oxidative/protease-driven barrier injury, and (b) supports microvascular tone and endothelial signaling that maintain neurovascular unit homeostasis [93–95]. Preclinical studies in acute brain injury settings and chronic neuroinflammation models further suggest that H_2_S reduces early permeability/edema and can limit inflammatory cell trafficking into the CNS, consistent with an overall barrier-protective phenotype [92]. Together, BBB stabilization likely synergizes with systemic immunomodulation to sustain a lower-inflammatory brain milieu. Crucially, the BBB stabilization mechanisms described herein likely function in concert with the peripheral immunoregulatory effects detailed in the “Immunoregulatory effects of hydrogen sulfide” section, collectively shaping the neurovascular inflammatory response.

### Protection of mitochondrial function and inhibition of inflammasomes

H_2_S exerts a concentration-dependent biphasic effect on mitochondrial bioenergetics and electron transfer. In experimental systems, low-to-moderate H_2_S concentrations (30 to 50 μM) have been reported to support electron handling via the mitochondrial sulfide oxidation unit (SQOR→CoQ), thereby alleviating the burden on Complex I and improving ATP synthesis efficiency. In contrast, higher concentrations (50 to 300 μM) can inhibit Complex IV and impair respiratory flux, highlighting the need to define a donor- and context-specific therapeutic window [96]. Beyond bioenergetics, H_2_S preserves mitochondrial stress tolerance by stabilizing thiol/redox balance, reducing mtROS generation, maintaining mitochondrial membrane potential, and limiting mitochondrial permeability transition pore (mPTP) opening; it may also promote mitochondrial quality control programs, including biogenesis and selective removal of damaged mitochondria [97–100].

**Fig. 8.**
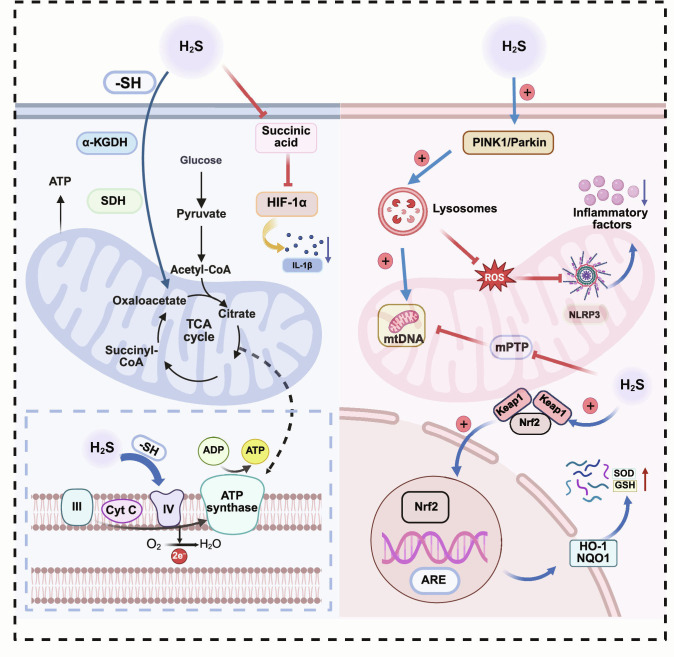
Mitochondria-centered mechanisms linking H_2_S to suppression of feed-forward neuroinflammation. H_2_S supports mitochondrial homeostasis through coordinated modules: it modulates bioenergetics by regulating TCA/respiratory functions (e.g., sulfhydration-sensitive control of key enzymes and electron-transfer capacity) [96,97]. Notably, H_2_S shows a biphasic exposure profile—low-to-moderate levels can support mitochondrial electron handling (including via sulfide oxidation-linked electron transfer), whereas higher levels may inhibit Complex IV and impair respiration [96]. In parallel, H_2_S activates antioxidant defenses via Keap1 modification and Nrf2-ARE signaling (e.g., induction of heme oxygenase-1/NAD(P)H quinone dehydrogenase 1 [HO-1/NQO1]) [101,102] and reinforces mitochondrial quality control (e.g., limiting mPTP opening and promoting PINK1/Parkin-dependent mitophagy) [98,103,148]. By reducing mitochondrial danger signaling (e.g., mtROS/mtDNA), these actions restrain NLRP3 inflammasome activation and downstream cytokine amplification [97,98] (created with BioRender).

In parallel, H_2_S activates antioxidant defenses through Keap1 modification and downstream Nrf2-dependent transcription [101,102]. By improving mitochondrial performance and redox buffering, H_2_S can reduce mtDNA leakage and other mitochondrial danger signals that couple metabolic stress to inflammasome activation, thereby restraining caspase-1-dependent cytokine maturation (e.g., IL-1β/IL-18) and reinforcing PINK1/Parkin-mediated mitophagy as an upstream brake on NLRP3 amplification [103,104]. Collectively, these effects provide an integrated mitochondria-centered explanation for how H_2_S suppresses feed-forward neuroinflammation (Fig. 8).

In summary, H_2_S mitigated neuroinflammation and shortened its duration through multitarget synergistic actions, including reshaping glial cell phenotypes (inhibition of NF-κB/MAPK, regulation of AMPK-mTOR, and enhancement of autophagic–phagocytic function), maintaining BBB integrity (inhibition of MMP-2/9, stabilization of tight junctions, and promotion of eNOS coupling and microcirculatory improvement), and coordinating mitochondrial homeostasis with NLRP3 inhibition (promotion of SQOR→CoQ electron transfer, activation of Nrf2-Keap1 signaling, and enhancement of PINK1/Parkin-mediated mitophagy).

## Advances in Hydrogen Sulfide-Based Therapeutic Strategies for NDDs

Building on immune modulation, BBB protection, and mitochondrial regulation, preclinical studies collectively support H_2_S as a multidomain intervention across AD, PD, and HD models (Table 1) [105–107]. Across diseases, the most consistent signals are attenuation of glia-driven inflammatory programs and mitigation of mitochondrial/oxidative stress, whereas effects on proteinopathy and synaptic function appear more model- and context-dependent. Nevertheless, heterogeneity in donor chemistry, dosing regimens, treatment windows, and delivery routes continues to limit cross-study comparability and remains a key barrier for translation.

**Table 1.** Representative anti-neuroinflammatory and disease-modifying actions of H_2_S across AD, PD, and HD models

Disease	Domain (primary axis)	Representative mechanism (one line)	Key outcomes (one line)	References
AD	Glia-driven inflammatory signaling (TLR4-p38/MAPK-NF-κB)	Suppresses TLR4/p38 (MAPK)/NF-κB activation in microglia	↓Proinflammatory cytokines/ROS; ↓neuroinflammation	[149–152]
AD	Proteinopathy (Aβ)	Reduces Aβ plaque deposition and related pathology	↑Cognition; ↓neuroinflammation	[55]
AD	Synaptic/neurotransmission support	AChE inhibition and synaptic facilitation (e.g., NMDA/LTP)	↑Cholinergic/synaptic function; ↑cognition	[153,154]
AD	Mitochondria/oxidative-nitrosative stress	Improves mitochondrial function/ATP and antioxidant capacity (e.g., ↑glutathione [GSH]; ↓iNOS/ONOO^-^)	↓Oxidative damage; ↓DAMP release	[152,154–156]
AD	Ca^2+^-linked signaling (supportive)	Modulates intracellular Ca^2+^ via cAMP/PKA	Contributes to anti-inflammatory signaling	[157]
PD	Microglial polarization/inflammatory signaling	Promotes M1→M2; inhibits P2X7R–MAPK and NF-κB pathways	↓Neuroinflammation; dopaminergic neuroprotection	[158,159]
PD	Nitrative stress and α-syn nitration	Slow-releasing H_2_S donor (GYY4137) limits nNOS/NO-driven nitrative stress	↓α-syn nitration; preserves TH^+^ neurons; improves motor performance	[160]
PD	Mitochondrial quality control	Protects respiratory chain; ↓ROS; stabilizes MMP; promotes biogenesis	↑Energy metabolism; ↓NLRP3 activation	[26,99,148,161]
PD	BBB integrity	Suppresses NF-κB signaling; reduces permeability	Protects CNS from peripheral inflammatory insults	[162]
HD	Microglia/inflammation resolution	Inhibits p38 MAPK/iNOS; pro-resolution dual-function compounds	↓Neuroinflammation; neuroprotection	[163]
HD	Peripheral–central immune crosstalk	Modulates neuro-immune communication; suppresses macrophage activation	↓Peripheral infiltration; ↓inflammation spread	[90,164]
HD	Oxidative stress-Nrf2 axis/mitochondria	Activates Nrf2/HO-1; ↑GSH; anti-ferroptosis; supports mitochondrial biogenesis	↓Oxidative damage; ↓inflammasome activation; ↑energy status	[102,165–168]
HD	Other (vascular/epigenetic)	eNOS up-regulation and epigenetic regulation	Improves vascular function; multiroute modulation	[93]

### Preclinical studies of hydrogen sulfide in different NDDs

Across diverse ischemia–reperfusion and inflammatory models, H_2_S exhibits a concentration-dependent (often U-shaped) profile, with cytoprotection at lower exposure but impairment at higher levels, underscoring the need for careful titration [108]. Studies showed that long-term sodium hydrosulfide (NaHS) reversed cognitive impairment and synaptic plasticity deficits in AD model mice, improved hippocampus-dependent contextual fear memory and novel object recognition, and promoted functional recovery [109]. Another report demonstrated that in APP/PS1 transgenic AD mice, dietary methionine restriction improved cognitive function by influencing endogenous H_2_S-generating pathways [110]. This effect may be associated with enhanced H_2_S production mediated by the transsulfuration pathway (CBS/CSE) and metabolic reprogramming of one-carbon/sulfur metabolism; however, causality remains to be further validated. To provide a parameter-informed overview of preclinical interventions, representative dose–exposure–effect–safety relationships of H_2_S-based treatments in AD and related cognitive impairment models are summarized in Table 2 [111,112].

**Table 2.** Representative dose–regimen–effect–safety profiles of H_2_S-based interventions in AD and related cognitive-impairment models

Model (type)	Intervention (donor; dose; route; duration)	Key mechanistic readouts (selected)	Behavioral outcomes (selected)	Safety/window (as reported)	References
Aβ (1–40) rat; 3xTg-AD mouse; STZ rat (subacute→chronic)	NaHS 0.25–1 mg/kg/day i.p.; and/or H_2_S-rich Tabiano water 12 ml/kg/day i.p.; 15 days–5 months	↓Aβ burden; ↓APP/tau phosphorylation; ↓oxidative/nitrosative stress; ↓TNF-α; ↓apoptosis; ↓JNK/p38/ERK MAPK activity	Improved Morris water maze performance	No signs of toxicity; body weight comparable between groups	[55,109]
STZ rat (diabetic cognitive impairment; chronic metabolic)	NaHS 30 or 100 μmol/kg/day (≈1.68 or 5.6 mg/kg/day) i.p.; 30 days	↓ER stress (GRP78, CHOP, cleaved caspase-12); ↑endogenous hippocampal H_2_S	Improved spatial/recognition memory (MWM, Y-maze, NOR)	100 μmol/kg/day showed no adverse effect on cognition in normal rats	[109]
β2-microglobulin (B2M) rat (subacute neuroinflammatory)	NaHS 30 or 100 μmol/kg/day (≈1.68 or 5.6 mg/kg/day) i.p.; 2 weeks	Restored hippocampal autophagic flux (↓p62; normalized autophagosome/lysosome balance)	Improved learning/memory (MWM, Y-maze, NOR)	No treatment-related toxicity or abnormal behavioral phenotypes reported	[111]
APP/PS1 mouse (chronic transgenic AD)	NaHS 2.8 mg/kg i.p., once daily; 28 days	↑Hippocampal H_2_S; restored LTP; prevented GluN2B down-regulation; normalized pCaMKII/pCREB and BDNF	Improved contextual fear memory and novel object recognition	No changes in pain threshold, locomotor activity, or anxiety-like behavior reported	[112]

In PD research, toxin-based models (1-methyl-4-phenyl-1,2,3,6-tetrahydropyridine [MPTP] and 6-hydroxydopamine [6-OHDA]) consistently indicate that H_2_S treatment can improve motor function and dopaminergic outcomes, in parallel with shifts toward anti-inflammatory glial programs, improved mitochondrial homeostasis, and restraint of inflammasome-associated signaling [113]. Notably, MPTP and 6-OHDA differ in lesion topography, mitochondrial liability, and time course; therefore, dosing regimens and exposure windows should be interpreted within each model rather than directly extrapolated across paradigms. A cross-model comparison of dosing regimens, exposure windows, and reported safety margins in PD and acute CNS injury models is summarized in Table 3.

**Table 3.** Dose–exposure–effect–safety profiles of H_2_S-based interventions in PD and acute CNS injury models

Disease/model	Model type	H_2_S donor and dose	Exposure regimen (route; timing)	Targeting emphasis	Key effects (selected)	Safety/toxicology notes (as reported)	References
PD (rotenone rat; 6-OHDA rat)	Toxin models; chronic→subacute	NaHS 1.68 or 5.6 mg/kg	i.p.; pretreatment and treatment; 3–4 weeks	Systemic donor; metabolic/oxidative/inflammatory modulation	↓Inflammation and microglial activation; ↓oxidative stress; improved motor/rotational behavior	Plasma H_2_S ↑~20% at 5.6 mg/kg; no notable systemic toxicity reported	[113]
PD (MPTP mouse)	Toxin model; acute/subacute	Inhaled H_2_S 40 ppm	Inhalation; 8 h/day for 7 consecutive days	Systemic gaseous delivery; mitochondrial/redox support	↑Antioxidant defenses; ↓apoptosis/gliosis; prevented dopaminergic neurodegeneration	Tolerable in this paradigm; no sustained respiratory depression reported at 40 ppm	[19]
Acute CNS injury (SCIRI rat)	Acute ischemia–reperfusion	GYY4137 50 mg/kg	i.p.; single injection 30 min before reperfusion	Cerebrovascular/BBB protection prominent	Improved neurological scores; ↓neuronal loss; improved neuroglia/BBB indices; ↓PANoptosis/neuroinflammation	No toxicity reported at 50 mg/kg in this acute setting	[169]
PD (MPTP mouse)	Toxin model; acute/subacute	GYY4137 12.5/25/50 mg/kg	i.p.; once daily; start 3 days before MPTP and continue 2 weeks after	Slow-releasing systemic donor; anti-nitrative/anti-inflammatory	↓α-syn nitration; ↓nitrative stress and inflammatory markers; preserved TH^+^ neurons; improved motor deficits	Safety margin suggested: 50 mg/kg GYY4137 alone showed no detectable behavioral impact	[170]

In HD studies, H_2_S intervention was found to alleviate oxidative stress and mitochondrial dysfunction caused by mHTT protein aggregation, improve motor coordination and grip strength in model animals, and partially delay disease progression [114]. Current evidence is largely derived from animal and cellular experiments, and the long-term safety and sustained efficacy of H_2_S still require validation in larger-scale studies.

### Hydrogen sulfide donor types and characteristics

H_2_S donors can be categorized by release kinetics and subcellular targeting, which together determine peak exposure, duration, and safety margins. Fast-releasing inorganic salts (e.g., NaHS, Na_2_S, and CaS) generate transient high peaks and are therefore mainly used in acute paradigms; however, careful titration is required because excessive exposure may inhibit Complex IV and compromise cellular respiration [115,116]. In contrast, slow-releasing organic donors (e.g., GYY4137) provide more sustained delivery and are generally better suited for chronic NDD models, where anti-inflammatory and neuroprotective effects have been reported [117]. Mitochondria-targeted donors such as AP39 (TPP^+^-conjugated) preferentially accumulate in mitochondria and can improve mitochondrial homeostasis and oxidative stress control at low doses, making them particularly relevant to NDD settings characterized by prominent bioenergetic failure [118].

Across models, therapeutic benefit is frequently observed within a lower exposure range, whereas poorly controlled or excessive delivery can shift the balance toward oxidative/nitrative stress and bioenergetic collapse, consistent with a narrow and potentially biphasic (U-shaped) exposure–response relationship [116]. Accordingly, donor class, dose, and treatment duration should be selected based on preclinical dose–exposure–response evidence rather than extrapolated across donor types; in many chronic paradigms, slow-releasing or targeted donors appear to offer more controllable exposure profiles than fast-releasing salts [113,116]. Naturally derived organosulfur precursors (e.g., diallyl trisulfide and related dietary compounds) may also increase H_2_S in vivo, but their release profiles and bioavailability show substantial interindividual variability [119].

Beyond exogenous donors, directional manipulation of H_2_S signaling by synthesis inhibition and the human observational evidence for endogenous dysregulation are summarized in Table 4, highlighting that both H_2_S deficiency and excess may be deleterious and underscoring the need for exposure monitoring and biomarker-guided titration when designing combination or hybrid strategies [120–122].

**Table 4.** Dose–response relationships and safety considerations of H_2_S signaling modulation

Category	Strategy/compound (direction)	Dose/exposure (preclinical or clinical)	Mode of action/targeting	Key effects (selected)	Therapeutic window/safety considerations	References
Donor (↑H_2_S)	NaHS (sodium hydrosulfide; fast-releasing)	In vivo: 5.6 mg/kg/day i.p.; 28 μmol/kg i.p.; 30–100 μmol/kg/day (various models). In vitro: 25–200 μM	Rapid-releasing inorganic donor; systemic exposure	Raises tissue/cellular H_2_S; improves cognition in AD/vascular dementia models with anti-inflammatory, antioxidant, and mitochondrial-protective signals	Biphasic profile: low μM/low mg·kg^−1^ ranges are typically neuroprotective; higher concentrations (≥100 μM in vitro) may be neurotoxic, supporting a narrow/U-shaped exposure–response	[120]
Inhibitor (↓synthesis)	AOA/AOAA (aminooxyacetic acid; CBS/CSE inhibitor)	8.75 mg/kg/day i.p. from postnatal day 75 to end-stage in SOD1^G93A ALS mice	Inhibits CBS and CSE; broad transaminase inhibition	↓H_2_S in brain/spinal cord; in female ALS mice: ~10-day lifespan extension and improved rotarod; ↓IBA1^+^ microglia; ↑GFAP^+^ astrocytes; limited benefit in males	No frank toxicity at tested dose, but off-target actions (transaminases; GABA metabolism) limit specificity; sex-dependent and modest efficacy suggests limited therapeutic margin	[121]
Human (observational)	Endogenous H_2_S dysregulation in ADRD patients	Observational cohort; plasma H_2_S metabolites measured (no exogenous compound)	Endogenous H_2_S overproduction signature (circulating pools)	↑Total plasma H_2_S, acid-labile sulfide, and bound sulfide in ADRD vs. controls; total H_2_S predicted ADRD (AUC ≈ 0.94) and correlated with cognition and white-matter lesion burden; mediation suggested ~49% of lesion–volume effect on cognition	Supports U-shaped biology: both excess and deficiency may be deleterious; motivates titration and exposure monitoring in H_2_S-modulating trials	[14]

### Combination therapy and hybrid molecule strategies

To enhance efficacy while reducing off-target toxicity, recent work has explored hybrid molecules and combination strategies that couple H_2_S release to established neurotherapeutics. In AD-oriented designs, H_2_S-memantine hybrids (e.g., Memit) and H_2_S-linked cholinesterase-inhibitor derivatives aim to retain symptomatic benefit while adding anti-aggregation, anti-inflammatory, and pro-homeostatic actions [123,124]. In PD, ACS84 (an H_2_S-L-DOPA hybrid) is a representative approach to mitigate dopaminergic dysfunction while buffering L-DOPA-associated oxidative and inflammatory stress [117]. Beyond CNS-specific drugs, conjugation or co-administration with non-steroidal anti-inflammatory drugs has been pursued to preserve COX inhibition while improving neuroprotection and potentially reducing gastrointestinal liability, which may be particularly relevant in stages dominated by neuroinflammation [125].

These considerations parallel the broader experience with repurposed metabolic/cardiovascular agents in PD, where both metformin and statins have been argued to exert stage- and context-dependent benefits versus risks, underscoring the importance of exposure-guided dosing and patient stratification [126,127].

However, translation of these hybrid/combination strategies remains constrained by PK/PD compatibility (matching release kinetics to target-organ exposure), the need to define a safe therapeutic window under potentially biphasic (U-shaped) H_2_S biology, and the lack of validated exposure/response biomarkers for dose titration. Representative agents, models, and mechanistic targets are summarized in Table 5 to provide an at-a-glance map for selecting candidate scaffolds and prioritizing endpoints in future preclinical and early-phase translational studies.

**Table 5.** H_2_S-based donors and hybrid molecules: representative mechanisms and targets in NDD models

Disease(s)	Agent/source	Biological model	Primary target(s)/mechanism (concise)	Key outcome (concise)	References
AD/PD/HD	AP39 (mitochondria-targeted H_2_S donor)	Human vein endothelial cells; SH-SY5Y neuroblastoma cells	Preserves mitochondrial function and limits oxidative stress	Improved mitochondrial resilience under stress	[22]
AD/PD/HD	NOSH-ASA (NO/H_2_S-releasing aspirin)	Human microglia; THP-1 cells	Suppresses NF-κB and p38 MAPK signaling; lowers TNF-α and IL-6 release	Anti-inflammatory signaling in immune cells	[171]
AD	Dietary/natural organosulfur compounds (food-derived precursors)	Transgenic AD mouse models	Supports endogenous H_2_S signaling; anti-apoptotic/anti-oxidative programs	Reduced neuronal injury in hippocampal CA1	[172]
AD	NaHS (fast-releasing H_2_S donor)	Aβ (1–40)-based AD rat model	Anti-apoptotic signaling in hippocampus	Attenuated Aβ (1–40)-induced CA1 apoptosis	[149]
AD	GYY4137 (slow-releasing H_2_S donor)	3xTg-AD mouse model	Persulfidation of GSK3β; reduces tau hyperphosphorylation	Lowered tau pathology in vivo	[173]
AD	H_2_S–memantine hybrid (Memit)	H9-derived neural stem cells	Reduces Aβ1–42 self-aggregation (and retains anti-excitotoxic framework)	Inhibited Aβ1–42 aggregation in vitro	[123]
AD	H_2_S-rivastigmine derivative (Derivative 1)	Neurons and microglia (culture)	AChE inhibition plus anti-inflammatory/anti-ROS signaling; induces autophagy	Reduced inflammation/ROS; limited Aβ fibril propagation	[124]
PD	NaHS (fast-releasing H_2_S donor)	6-OHDA-induced PD rat model	Modulates oxidative stress/inflammation; alters leptin signaling and autophagy flux (as reported)	Improved PD-related outcomes with mechanism context-dependent	[170,174]
PD	ACS84 (H_2_S-L-DOPA hybrid)	6-OHDA-induced PD model	Limits oxidative stress and dopaminergic neuron loss during L-DOPA therapy	Reduced TH^+^ neuron loss and dopamine depletion; improved motor phenotype	[175]
HD	NaHS (fast-releasing H_2_S donor)	HD-like rat model	Up-regulates CBS expression; anti-apoptotic/anti-inflammatory/antioxidant programs	Improved pathological readouts in vivo	[176]

**Fig. 9.**
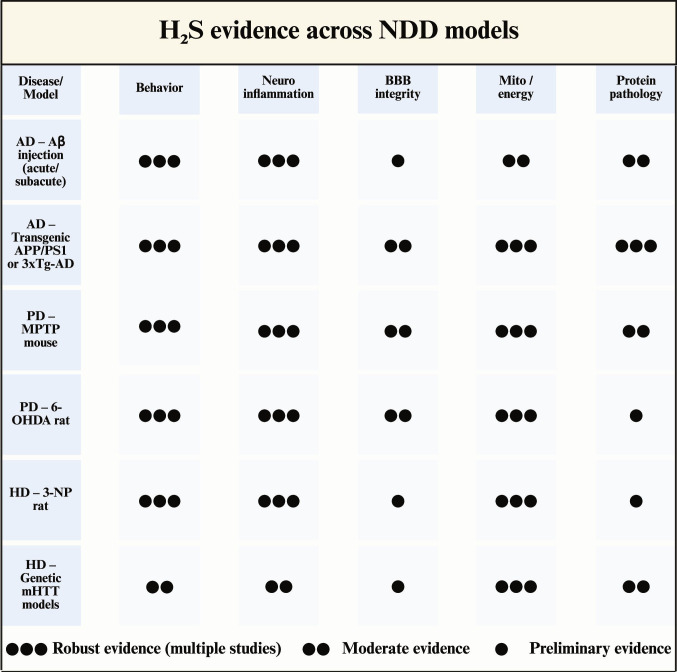
Semiquantitative evidence matrix of H_2_S-based interventions in preclinical models of NDDs.

To synthesize heterogeneous preclinical outcomes across NDD models, Fig. 9 provides a semiquantitative evidence matrix that compares the consistency of reported H_2_S-mediated benefits across core endpoint domains.

Rows represent commonly used model-disease combinations (AD: Aβ injection; APP/PS1 or 3xTg-AD transgenic models; PD: MPTP and 6-OHDA toxin models; HD: 3-NP toxin and genetic mHTT models) [110,113]. Columns summarize outcome domains assessed in the literature, including behavior, neuroinflammation, BBB integrity, mitochondrial/energy homeostasis, and protein pathology; the domain definitions are aligned with the corresponding sections and Tables 1 to 5 (see also Fig. 4, 7, and 8 for mechanistic context [67,128,129]). Filled circles indicate the relative strength/consistency of reported effects for each domain (created with BioRender).

## Summary and Perspectives

### Synergistic suppression of neuroinflammation in NDDs by hydrogen sulfide

NDDs are driven by a self-perpetuating cycle of neuroinflammation, characterized by the aberrant activation of resident immune cells, BBB disruption, and mitochondrial dysfunction. H_2_S breaks this cycle through a multitarget mechanism: it modulates microglial and astrocytic phenotypes toward reparative states, preserved BBB integrity to limit peripheral infiltration, and restores mitochondrial quality control via mitophagy. While existing evidence confirmed H_2_S as a potent immunomodulator, future work must move beyond phenomenology to map the precise spatiotemporal kinetics of these effects in distinct disease stages.

### Crosstalk within the gaseous signaling molecule network and therapeutic prospects

Therapeutic outcomes depend not on H_2_S alone, but on its integration into a dynamic “gasotransmitter interactome” involving NO, CO, and H_2_. These molecules engage in complex feedback loops that fine-tuned inflammation and redox balance [130]. A critical future direction is to decipher the quantitative logic of this network. We suggest that researchers employ systems biology approaches and mathematical modeling to predict how H_2_S donors influence the fluxes of NO and CO under pathological conditions. Building upon the foundation of H_2_S monotherapy, the development of hybrid donors and codelivery nanoplatforms (e.g., those releasing H_2_S and NO in defined ratios) represents a robust strategy to harness their synergistic neuroprotective potential while minimizing off-target effects.

### Gut–brain axis-mediated modulation of hydrogen sulfide synthesis signaling in NDDs

Peripheral H_2_S, particularly from the gut microbiota, emerges as a remote regulator of central neuroinflammation. While physiological H_2_S maintains the mucosal barrier, dysbiosis-driven excessive H_2_S is implicated in promoting amyloid and tau pathology [28,131,132]. Future translational research will focus on “drugging” this axis. Concrete strategies include the following: (a) dietary interventions to modulate sulfur-containing amino acid intake; (b) next-generation probiotics engineered to regulate luminal H_2_S levels; and (c) gut-restricted H_2_S scavengers to prevent epithelial leakage and subsequent neuroimmune activation [133–136]. Validating whether these peripheral interventions could modify central markers of neurodegeneration would be a key step toward noninvasive therapies.

### Spatial multiomics and biomarker-guided precision hydrogen sulfide synthesis therapies

A major hurdle in translation is the heterogeneity of neuroinflammation. Future studies must leverage spatial transcriptomics and metabolomics to dissect how H_2_S signaling varies across distinct brain regions and within specific niches (e.g., around amyloid plaques vs. neurofibrillary tangles) [137]. By integrating these spatial maps with pharmacological data, researchers can identify specific “H_2_S-responsive” cell subpopulations. Clinically, this necessitates the validation of fluid biomarkers (e.g., sulfane sulfur levels in plasma or CSF) that correlate with brain H_2_S status. Such biomarkers are essential for patient stratification, ensuring that H_2_S-based therapies are targeted specifically to individuals with demonstrable sulfur metabolism defects [138–141].

### Challenges and strategies for the clinical translation of hydrogen sulfide

Despite preclinical success, clinical translation remained hindered by the “delivery–monitoring gap”. Its biological effects are highly concentration-dependent: protective at low concentrations (30 to 50 μM) but cytotoxic at higher levels (50 to 300 μM). This underscores the need for in situ dynamic monitoring systems to enable precise regulation of local concentrations. However, real-time H_2_S detection technologies capable of penetrating the BBB remain underdeveloped, highlighting the need for innovative monitoring tools such as reversible fluorescent probes and miniaturized biosensors.

Beyond CNS delivery, systemic inflammatory states and cardio-metabolic comorbidities can reshape neuroimmune tone and treatment response. For example, recent literature highlights the complex endocrine–immune interplay (e.g., involving arginine vasopressin) in systemic pathologies like COVID-19, suggesting that peripheral mediators may considerably confound inflammatory trajectories in NDD patients [142]. Likewise, the evaluation of pleiotropic anti-inflammatory candidates (such as ursolic acid) emphasizes the critical need for rigorous PK/PD and safety validation before repurposing broad-spectrum agents for chronic neurological indications [143]. Moreover, endocrine–metabolic variability and concomitant medications can modify systemic inflammatory biomarkers (e.g., hyperbilirubinemia-related attenuation of COVID-19 metabolic disturbances and drug-associated prolactin changes) [144,145], and vascular inflammatory markers such as lipoprotein-associated phospholipase A2 may reflect cardiometabolic status relevant to neurovascular outcomes [146].

To enhance drug delivery efficiency, CNS-targeted delivery strategies are being explored, including ApoE (apolipoprotein E)-modified nanoparticles and thiol-activated prodrugs, which aim to increase H_2_S bioavailability in the brain, enable precise dosing, and minimize peripheral toxicity. Future research should prioritize stimulus-responsive nanocarriers (e.g., pH-sensitive or ROS-triggered release) that can traverse the BBB and release H_2_S exclusively at sites of neuroinflammation, thereby widening the therapeutic window [22]. Additionally, there is an urgent need for noninvasive imaging modalities (such as H_2_S-specific PET tracers or MRI probes) capable of quantifying brain H_2_S levels in real-time patients.

### Limitations and confounders

Several factors limit cross-study comparability and may confound interpretation of H_2_S efficacy across NDD models. First, donor chemistry and release kinetics differ substantially, and many studies lack quantitative brain exposure measurements, making dose translation and safety margins uncertain. Second, outcomes are influenced by model choice (toxin vs. transgenic), disease stage, sex, age, and comorbid metabolic or vascular conditions, which can show context-dependent or even bidirectional effects and are inconsistently reported [126,127]. Third, microbiome composition, diet, and housing conditions can modify endogenous sulfur metabolism and inflammatory tone, potentially contributing to between-laboratory variability. Future work should adopt standardized reporting of exposure proxies and key biological covariates, and incorporate biomarker-guided stratification to improve translational relevance.

In summary, H_2_S holds immense promise as a multitarget modulator of neuroinflammation. To translate this potential into clinical reality, the field will transition from describing molecular mechanisms to engineering precise delivery systems and valid biomarkers. By integrating crosstalk modeling, gut–brain axis modulation, and spatial precision medicine, future research can overcome current bottlenecks and establish H_2_S-based interventions as a cornerstone of NDD therapy.
